# Analysis of Marijuana (*Cannabis sativa* L.) Cuttings: Morphological and Colorimetric Traits as Predictors for Optimization of Vegetative Reproduction

**DOI:** 10.3390/plants15030440

**Published:** 2026-01-31

**Authors:** Laura G. A. Espósito, Camila Rodrigues, Pedro Pereira, Heitor Mancini Teixeira, Derly Silva

**Affiliations:** 1 Graduate Program in Plant Science, Department of Agronomy, Federal University of Viçosa (UFV), Avenida Peter Henry Rolfs, Viçosa 36570-900, Brazil; derly@ufv.br; 2 Undergraduate Program in Agronomic Engineering, Department of Agronomy, Federal University of Viçosa (UFV), Avenida Peter Henry Rolfs, Viçosa 36570-900, Brazil; camila.f.rodrigues@ufv.br (C.R.); pedro.f.oliveira@ufv.br (P.P.); 3 Graduate Program in Agroecology, Department of Soils, Federal University of Viçosa (UFV), Avenida Peter Henry Rolfs, Viçosa 36570-900, Brazil; heitor.teixeira@ufv.br

**Keywords:** adventitious rooting, apical meristem, clonal propagation, hemp, logistic regression, predictive modeling, propagule performance, rhizogenesis, rooting probability, stem cutting

## Abstract

Marijuana (*Cannabis sativa* L.) has a great economic potential due to its phytotherapeutic properties. Its propagation, however, faces numerous challenges due to the limited availability of standardized technical protocols for the crop. Vegetative propagation represents a, or even the, viable method for multiplying the genetically identical individuals while preserving their phytochemical profile, at lower costs and with shorter production times. This study investigated the morphological and colorimetric attributes associated with vegetative propagation success, aiming to develop sustainable cultivation strategies. Four cutting lengths (5, 10, 15 and 20 cm) were evaluated after 21 days of rooting, considering fresh mass, basal diameter, presence of apical meristem, number of root primordia, root length, and foliar and stem color parameters. Logistic regressions indicated that longer cuttings (*p* = 0.0101), greater fresh mass (*p* = 0.073) and the presence of apical meristem (*p* = 0.065), as well as greener leaves (*p* = 0.089), were positively associated with rooting probability (*p* < 0.10). Positive correlations between morphological and colorimetric variables were confirmed by Principal Component Analysis, with the first two principal components explaining 31.2% of the total variance in the dataset. The results provide support for the development of more efficient and low-cost vegetative propagation protocols, promoting uniformity and autonomy in local cutting production of marijuana.

## 1. Introduction

The botanical species *Cannabis sativa* L., with its stigmatized common name “marijuana” refers to an annual herbaceous plant belonging to the family Cannabaceae and originating from Central Asia. It has been widely cultivated and used by ancient civilizations for thousands of years for various purposes: therapeutic, industrial, nutritional, and spiritual [1], as it continues to occur today. In addition to its recognized versatility of uses, it stands out for its high phenotypic plasticity and genetic diversity, which facilitate its adaptation to diverse edaphoclimatic conditions and make it a strategic resource for sustainable agriculture in different socio-environmental contexts [2]. Nevertheless, the establishment and expansion of its cultivation face challenges related to propagation, especially for purposes requiring the maintenance of an agronomic and phytochemical profile, such as phytotherapy [3,4,5].

Although propagation by seed is possible, sexual reproduction results in high variability among plants, hindering phytochemical standardization and, consequently, the quality of therapeutic products [4]. Thus, propagation by cuttings (a vegetative reproduction technique that uses parts of the mother plant to generate new, genetically identical individuals) is the preferred method for preserving genotypes, reducing cultivation time, and increasing uniformity among individuals [6,7], particularly with respect to the profiles and proportions of cannabinoids, terpenes, and other medicinal compounds. This technique is currently the only feasible method in countries such as Brazil, where seeds “do not exist” and are financially unviable for constant importation. Therefore, the success of vegetative propagation is an essential aspect of cultivation.

Previous studies have emphasized several factors such as cutting position on the mother plant, the number of leaves, and the use of phytohormones as determinants of rooting [7]. However, the other variables still require investigation, especially under tropical conditions and with a view toward production for associative/community cultivation, ranging from domestic to industrial scales, in response to the continuously growing demand for marijuana-derived products. This demand is driven by the emergence of new markets from political/legal perspectives, as an increasing number of countries and states have adopted legislation enabling medicinal (and recreational) use, as well as by the development of new products across multiple sectors [8]. The advances in scientific research and the gradual shift in regulatory frameworks are strengthening the recognition of the species as a relevant resource for the economy, the environment, and public and individual health, within the scope of the Brazilian Unified Health System (SUS). In Brazil, patient associations, social movements, researchers, and farmers have played a central role in democratizing access, evidencing the need to establish and disseminate low-cost, high-efficiency cultivation protocols grounded in scientific rigor.

In this context, this study is based on the hypothesis that morphological and colorimetric attributes of cuttings significantly influence on rooting success and may serve as indicators for selecting of more efficient propagules. In this respect, the objective was to contribute to the understanding of factors relevant to the optimization of marijuana cutting propagation collaborating in the future development of phytotechnical protocols for cultivation, evaluating the morphological attributes of propagules such as length, fresh mass, basal stem diameter, and the presence of the apical meristem; and foliar and stem colorimetric parameters (CIE-LAB), in their relationship with rhizogenesis.

## 2. Materials and Methods

### 2.1. Location

The experiment was conducted in a medical marijuana patient association (coordinates 22°25′44″ S, 43°24′08″ W, and 624 m in altitude), located in the rural area of the municipality of Paty de Alferes, state of Rio de Janeiro, Brazil. According to the Köppen climate classification, the region presents a Cfa climate, characterized as humid subtropical, with hot and humid summers and mild winters, as defined for southeastern Brazil [9].

### 2.2. Plant Material

The mother plants of marijuana (*Cannabis sativa* L.) were established from a genetically stabilized cultivar (‘Doctor’). This cultivar is characterized by high Cannabidiol (CBD) biosynthetic potential, with average concentrations of approximately 33 mg mL^−1^ in the oil formulations distributed by the association. The seeds were sown in trays containing a substrate composed of 30% coconut husk, 25% organic compost, 20% sand, 15% commercial seedling substrate (Carolina Soil^®^), and 10% composted cattle manure. Mother plants were maintained in the vegetative stage under an 18 h photoperiod (natural and supplemental artificial light), without any additional environmental control or physical (structural) protection in cultivation, during the 12 weeks preceding cutting collection, under local climatic conditions characterized by a mean daily maximum temperature of 28.2 °C and a mean daily minimum temperature of 18.2 °C. During the same period, mean relative humidity was 79.4%, and mean daily minimum relative humidity was 53.17%, according to data obtained from the National Institute of Meteorology (INMET) [10].

### 2.3. Experimental Design

The experiment was conducted using a completely randomized design (CRD). Cuttings were randomly selected along the shoots of each mother plant, without positional preference, to minimize bias associated with node position or shoot vigor. Subsequently, all cuttings were randomly distributed within the propagation area at the time of establishment.

**Figure 1:**
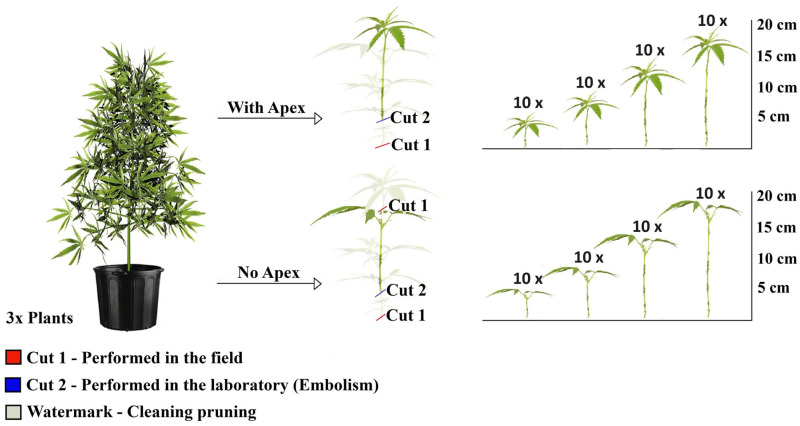
Experimental design of the rooting of marijuana cuttings (*Cannabis sativa* L.), obtained from three mother plants, with and without the shoot apex, at lengths of 5, 10, 15 and 20 cm, with 10 replicates per treatment. The watermark on the discarded plant material represents sanitation pruning.

Treatments consisted of cuttings of four different lengths: 5, 10, 15 and 20 cm. For each length, cuttings were evaluated in two independent groups, defined by the presence or absence of the apical stem meristem. Each treatment included 10 replications. Cuttings were obtained from three mother plants, with 80 cuttings collected from each, resulting in a total of 240 observations (Figure 1).

### 2.4. Procedures

The cuttings were collected from the mother plants with an average excess of 3 cm, which was discarded to eliminate the region at risk of vascular system embolism. The first cut (red) was performed in the field to obtain the propagule, whereas the second cut (blue) was carried out in the laboratory after the cleaning prune (indicated as a watermark), resulting in cuttings with one fully developed pair of leaves. The final length of the cuttings intended for rooting was defined after measuring the variables of interest.

The rooting was conducted in a laboratory adapted as a propagation room, equipped with a window in direct contact with the external environment and therefore subject to local climatic variation. During the rooting period, environmental conditions were characterized by a mean daily maximum temperature of 31.1 °C, a mean daily minimum temperature of 19.5 °C, a mean daily relative humidity of 81.82% and a mean daily minimum relative humidity of 50.22%, according to data from the National Institute of Meteorology (INMET) [10]. Artificial lighting was provided by white-spectrum LED lamps set to a photoperiod of 18 h, without control or monitoring of light intensity at canopy level. Cuttings were placed in containers filled with the previously described substrate (Section 2.2). The irrigation was performed daily using a manual sprayer until the substrate reached field capacity. During the first week after establishment, cuttings also received daily foliar misting to minimize initial dehydration.

Considering the time required for rhizogenesis, the experiment was conducted over 21 days, with photoperiod (18 h) as the only controlled environmental variable. At the end of the experimental period, the substrate was removed using distilled water for photographic documentation of the propagules and for measurement of the variables of interest (Table 1). Propagules were considered as rooted when they presented countable root primordia on the stem tissue.

### 2.5. Colorimetry

The coloration of plant tissues was evaluated using a digital colorimeter (Color Reader CR-10, Konica Minolta Optics), applying the CIE-LAB system (Commission Internationale de l’Éclairage), in which “L” values indicate luminosity (0 = black; 100 = white), “A” values express variation between green (negative values) and red (positive values), and “B” values represent variation between blue (negative values) and yellow (positive values), as shown in Appendix A.

The L, A, and B parameters were used to determine the relative coloration of the leaf and stem epidermis of each cutting. Additionally, their variations at the end of rooting and the relationship with rhizogenesis success were evaluated.

### 2.6. Statistical Analysis

Statistical analyses were performed using the R software (Version 4.3.3) [11], through the integrated environment RStudio (Version 2026.01.0+392) [12]. Before model fitting, exploratory data analysis was performed to identify outliers, assess data distribution, and verify the suitability of the variables for the selected statistical approaches. The adequacy of the data to the statistical models and the validation of each model were tested by simulation-based residual diagnostics, using the DHARMa package, which indicated no violations of model assumptions: normality, homoscedasticity, and independence of residuals (Appendix B).

A significance level of 10% (α = 0.10) was adopted, applied across all inferential analyses, considering environmental variations during the assays—particularly temperature and relative humidity—which are known to influence physiological rooting processes [13]. Given the semi-controlled propagation conditions, this threshold was intentionally selected to reduce Type II error and to improve sensitivity to biologically meaningful effects, thereby enhancing the detection of physiologically relevant patterns and trends.

Continuous variables were analyzed using parametric mean *t*-tests when assumptions were met, and categorical or binary rooting responses were evaluated through simple and multiple logistic regression models, which are statistically more appropriate for the structure of the data.

Correlation analyses were performed to evaluate the strength and direction of associations between phytotechnical traits of propagules and rooting-related variables. These relationships were assessed using Spearman’s rank correlation coefficient, chosen due to the non-normal distribution of several variables and the presence of ordinal and proportional data. The results were summarized in a Spearman correlation matrix and further explored through Principal Component Analysis to identify multivariate patterns and trait associations.

The alignment between the research objectives, the explanatory variables associated with the phytotechnical quality of propagules, the response variables related to rooting, and the statistical methods appropriate to the nature of the data ensures a coherent approach to the assessment of vegetative propagation and guarantees methodological rigor. The relationship among objectives, variables, and methods was systematized into three analytical axes: morphometry, colorimetry, and correlation analyses, as presented in Table 2.

## 3. Results

All findings are explicitly presented as observations obtained under the experimental conditions tested.

### 3.1. Morphometry

Morphometry, the quantitative analysis of the dimensions, shapes, and proportions of plant organs, enables the characterization of phenotypic variations related to growth, development, and responses to environmental conditions or management practices. In this study, morphometric analysis was used to quantify variation in propagule dimensions and to identify size-related differences associated with rooting probability, thereby supporting the evaluation of cutting performance in relation to the efficiency of vegetative propagation.

To assess the influence of morphological attributes on rhizogenesis, easily measurable and low-cost phytotechnical traits were analyzed, including cutting length, fresh mass, basal stem diameter, and the presence of the apical stem meristem. These attributes were selected for their practical relevance and direct applicability in propagation management.

The preliminary morphometric analysis, based on descriptive statistics, revealed patterns in rhizogenesis as a function of cutting length (Table 3). Increasing cutting length promoted progressive increases in fresh mass and stem basal diameter, indicating greater structural development of longer propagules, as expected, as well as consistent improvements in rhizogenesis-related variables. Statistical significance among treatments was evaluated according to the methods described in Section 2.6, and the results are presented in the following sections.

#### 3.1.1. Cutting Length and Rooting Success

**Figure 2:**
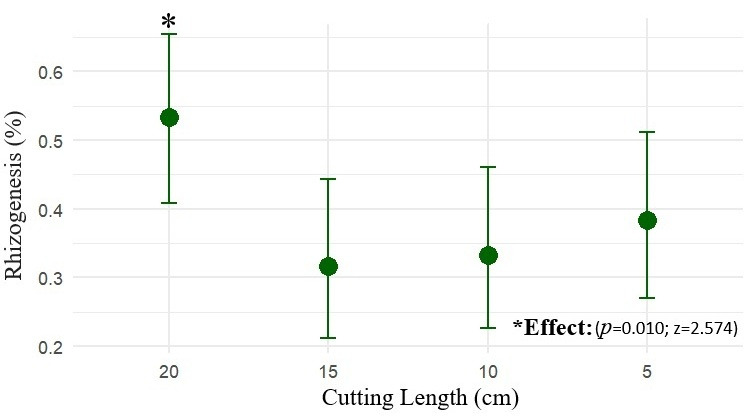
The mean rooting probability (%) of marijuana cuttings (*Cannabis sativa* L.) as a function of propagule length (5, 10, 15 and 20 cm), with 90% confidence intervals (CI_90_).

Propagule length influenced the probability of marijuana rooting. Propagules measuring 20 cm exhibited the highest rooting rate, estimated at 53.3% (CI_90_: 42.7–63.6%), a value higher than the other treatments (z = 2.574; *p* = 0.010). Propagules of 15 cm (z = –2.379; *p* = 0.017) showed estimated rooting rates of 31.7% (CI_90_: 22.7–42.2%), those of 10 cm (z = –2.194; *p* = 0.028) had rooting rates of 33.3% (CI_90_: 24.2–44.0%), and those of 5 cm (z = –1.642; *p* = 0.100) exhibited rooting of 38.3% (CI_90_: 27.7–49.0%), not differing statistically from one another, as shown in Figure 2.

#### 3.1.2. Cutting Fresh Mass and Rooting Success

**Figure 3:**
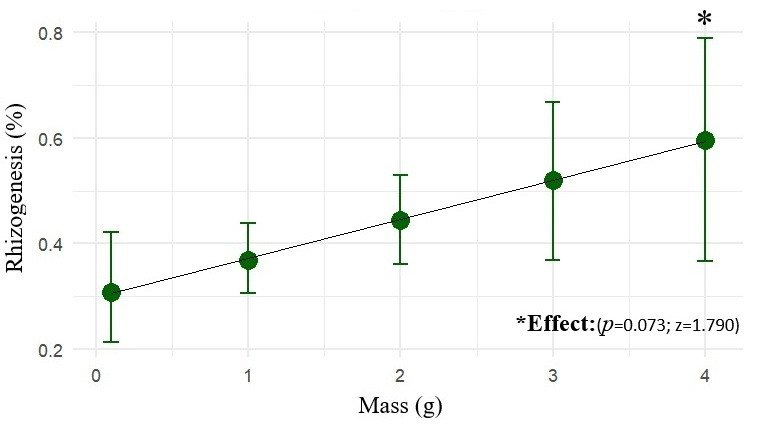
The mean rooting probability (%) of marijuana cuttings (*Cannabis sativa* L.) as a function of fresh mass (0–4 g), with 90% confidence intervals (CI_90_).

The fresh mass of the propagule positively influenced rooting probability. Within the evaluated range (up to 4 g), heavier propagules exhibited a greater odd of root formation (z = 1.790; *p* = 0.073), as shown in Figure 3. Thus, the fresh mass is confirmed as a relevant criterion for the selection of more promising propagules for marijuana cuttings.

#### 3.1.3. Basal Stem Diameter and Rooting Success

The diameter of basal propagule did not significantly affect rooting probability (*p* = 0.521). The estimated coefficient (β = 0.085) indicates that each additional millimeter in diameter increases the chance of rooting by approximately 9% (OR = 1.09; CI_90_: 0.91–1.30 mm), an interval that includes 1, confirming the absence of a statistical effect. Thus, within the observed range of variation, from 2 to 7 mm, basal stem diameter was not a relevant predictor of rooting success and therefore does not constitute a meaningful criterion for propagule selection.

#### 3.1.4. Apical Stem Meristem and Rooting Success

**Figure 4:**
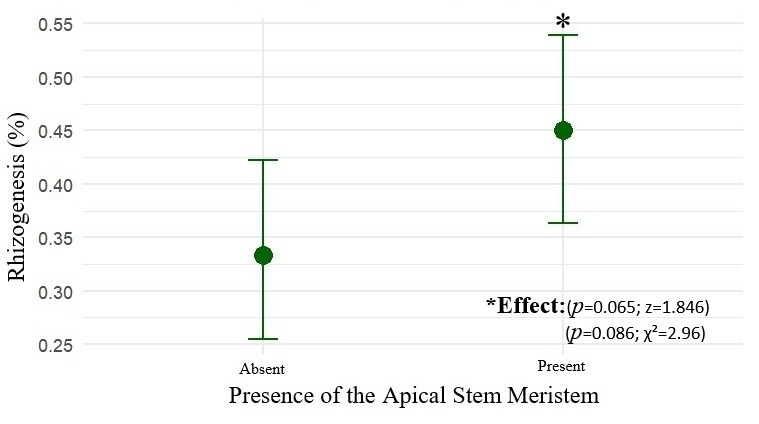
The mean rooting probability (%) of marijuana cuttings (*Cannabis sativa* L.) as a function of the presence or absence of the apical stem apex, with 90% confidence intervals (CI_90_).

The presence of the apical stem meristem had a positive influence on rooting probability (z = 1.846; *p* = 0.065). Cuttings without an apex exhibited an estimated rooting probability of 33.3% (CI_90_: 26.7–40.7%), while those with an apex reached 45.0% (CI_90_: 37.7–52.5%), as shown in Figure 4.

The logistic model indicated an odds ratio (OR) of 1.63 (CI_90_: 1.02–2.62), demonstrating that the presence of the apex increases rooting probability by up to 63% with 90% confidence. Results from Fisher’s test (*p* = 0.085) and the chi-square test (χ^2^ = 2.96; df = 1; *p* = 0.086) corroborate this finding. Thus, maintaining the apical meristem constitutes a relevant criterion for selection of propagules with higher vegetative propagation potential.

### 3.2. Colorimetry

Colorimetric analysis enabled the quantification of epidermal color variables for leaves and stems of the propagules, with the CIE-LAB system that provides the parameters of luminosity (L) and green–red (A) and blue–yellow (B) hues, aiming to assess their physiological and phytotechnical quality. These metrics are related to cutting vigor and nutritional status, reflecting their rooting probability. Thus, colorimetry is a useful tool to evaluate the performance of propagules in cutting propagation.

#### 3.2.1. Variation in Cutting Color and Rooting Success

**Figure 5:**
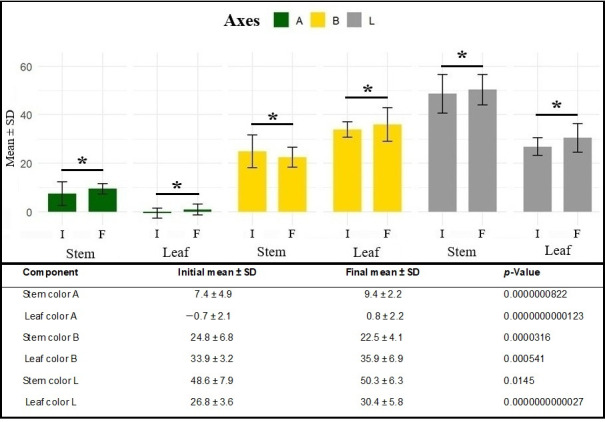
Initial and final mean ± standard deviation (SD) values of color components along the A, B, and L axes of the CIE-LAB system in stems and leaves during marijuana cutting propagation (*Cannabis sativa* L.). * indicates statistically significant at *p* < 0.10.

Comparative mean *t*-tests between initial color parameters (day of excision from the mother plant) and final parameters (21 days after cutting) revealed significant changes in all color components for both analyzed tissues (*p* = 0.0145 to *p* = 2.7 × 10^−12^). Mean values increased along the A axis in both stems and leaves, indicating a shift from green toward reddish hues. Along the B axis, stems showed reduced mean values, reflecting decreased yellow tones, whereas leaves exhibited increased values, consistent with yellowing or chlorosis of the leaf blade. Along the L axis, both tissues showed higher values at the end of the rooting period, indicating increased tissue lightness (Figure 5).

The changes observed in leaf and stem colorimetric parameters highlight the relevance of such measurements for inferring the physiological status of the propagules during the rooting process and, consequently, their relationship with rhizogenesis. The variations detected at the end of the 21 days likely reflect physiological responses inherent to rhizogenesis and the senescence under suboptimal conditions, rather than intrinsic traits suitable for propagule selection at the time of cutting. As the analysis focuses on temporal variation, these results are therefore presented as descriptive indicators of physiological dynamics, contributing to the interpretation of rooting performance without implying direct practical applicability for initial selection decisions.

#### 3.2.2. Cutting Color and Rooting Success

The association between initial colorimetric parameters of leaf and stem epidermis (day of cutting from the mother plant) and rooting success was evaluated using simple logistic regression models (for each color component individually) and multiple logistic models (assessing the combined association of L, A and B components) as predictors of rooting in cutting propagation (Table 4).

In the simple models, only the A component of leaf color showed a significant effect (*p* = 0.089), with an estimate lower than 1 (0.870; CI_90_: 0.752–0.985), indicating that lower values for this component—that is, a shift along the axis toward greener coloration—are associated with a higher probability of rooting. For the remaining leaf and stem parameters, no significant effects on rhizogenesis were observed (*p* > 0.21).

In the multiple models, statistically relevant effects were observed only for the leaf color components: results for the A axis showed a significant effect on rooting (estimate = 0.727; CI_90_: 0.590–0.874; *p* = 0.008), indicating that lower values for this parameter—i.e., greener coloration—are associated with a higher probability of rooting; for the B axis, significance (estimate = 0.894; CI_90%_: 0.800–0.985; *p* = 0.077) suggests that lower yellow intensity favors rooting. Leaf luminosity (L) showed no significant effect. The multiple models for leaf color resulted in a lower AIC (320) compared with the simple models (322–325), suggesting better model fit.

None of the stem color components exhibited a significant association with rooting, indicating no effect of stem color on rooting probability, corroborating the simple model results.

These findings indicate that leaf color—particularly parameters related to hue (A and B axes)—may be used as a nondestructive indicator of rooting potential in cuttings, whereas stem coloration did not demonstrate predictive relevance in this context.

### 3.3. Morphocolorimetric Correlations

Correlation analyses allowed the identification of relationships between morphological and colorimetric attributes and their association with propagule rooting success, a fundamental step for understanding the magnitude and direction of associations among variables. The identification of significant correlations supported the selection of morphometric and colorimetric indicators as robust predictors and the refinement of explanatory and predictive models, strengthening analytical rigor.

The Correlation Matrix, containing all morphological and colorimetric variables, was evaluated using Spearman’s coefficient, a nonparametric method suitable for asymmetric distributions. Principal Component Analysis was then applied to transform potentially correlated variables into independent components capable of retaining the greatest portion of explained variance. This multivariate approach enabled visualization of covariation patterns and identification of predictors of rhizogenesis in the propagules.

#### 3.3.1. Correlation Matrix

**Figure 6:**
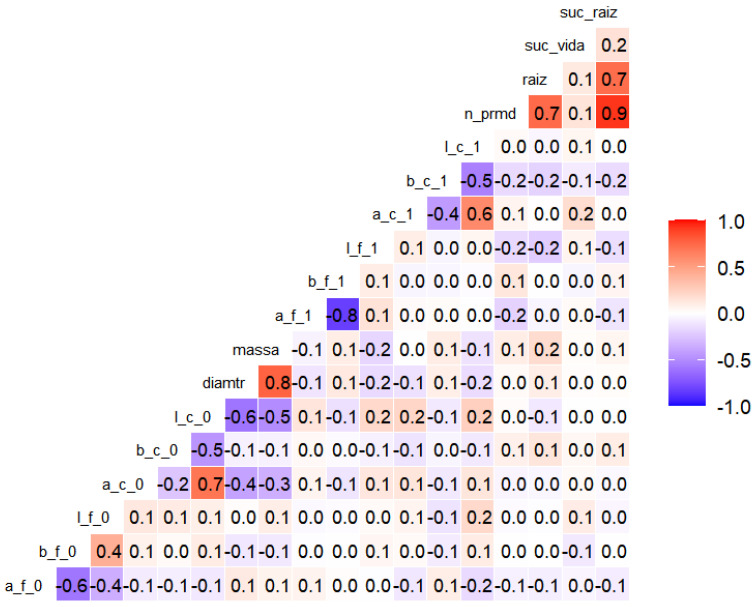
Heatmap of Spearman correlations (α = 0.10) between morphometric variables of the cuttings: mass, diameter, number of primordia (n_prmd), and length of adventitious roots (raiz); and colorimetric variables (components a, b, l) of leaves (f) and stems (c) at the initial (0) and final (1) times of the experiment with marijuana cuttings (*Cannabis sativa* L.). Red cells indicate the stronger positive correlations, whereas blue cells represent negative correlations. The coefficients correspond to Spearman’s ρ values.

The correlation analysis (Figure 6) revealed the expressive relationships between the morphological and colorimetric attributes of the propagules. A strong positive association was observed between rooting success and the number of root primordia (ρ = 0.9); between rooting success and the length of adventitious roots (ρ = 0.7); and between the number of root primordia and the length of adventitious roots (ρ = 0.7). Basal diameter showed a high correlation with fresh mass (ρ = 0.8).

Among the colorimetric variables, negative correlations were found between leaf A and B axes at the final timepoint (ρ = −0.8), which were also observed as moderate negative correlations at the initial timepoint (ρ = −0.6). The positive correlations were noted between the A axis and luminosity (L) at the initial (ρ = 0.7) and final (ρ = 0.6) evaluations.

Overall, the correlations identified between morphological vigor parameters and colorimetric parameters in cutting rhizogenesis may serve as nondestructive indicators of the physiological performance of propagules.

#### 3.3.2. Principal Component Analysis (PCA)

**Figure 7:**
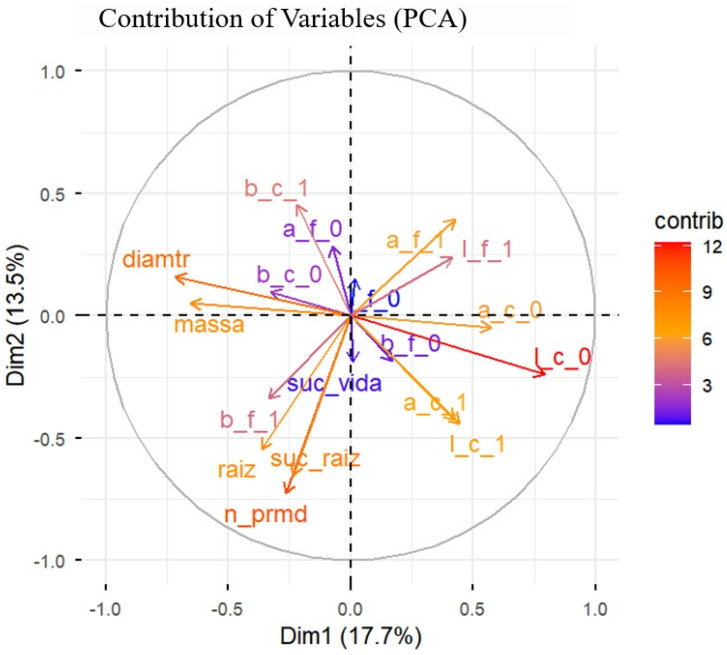
Principal Component Analysis, representing the contribution of the variables to the first two principal components (Dim1 and Dim2), which together explain 31.2% of the total data variance. The morphometric variables of the cuttings: mass, diameter, number of primordia (n_prmd), and length of adventitious roots (raiz); and the colorimetric variables (a, b, l) in leaves (f) and stems (c) at the initial (0) and final (1) times. The coloring of the vectors indicates the degree of individual contribution of each variable evaluated in marijuana (*Cannabis sativa* L.) cuttings to the structure of the principal axes.

The PCA synthesized the variability among all morphological and colorimetric attributes of the propagules (Figure 7). The first two dimensions jointly explained 31.2% of the total variation in the data (Dim1 = 17.7% and Dim2 = 13.5%), indicating that rooting success variability was partially captured by the measured traits and likely influenced by additional unmeasured or uncontrolled factors. All results are therefore interpreted within the context of the low-control, suboptimal conditions under which the experiment was conducted, inherent to the real-world propagation system.

## 4. Discussion

### 4.1. Morphometry

#### 4.1.1. Cutting Length and Rhizogenesis

The cutting length played a decisive role in the rhizogenic performance of Marijuana, especially in the genotype tested as 20 cm cuttings exhibited significantly higher rooting percentage compared with shorter propagules. These results indicate that cutting length acts as an integrative morphological factor for the success of vegetative propagation, insofar as longer cuttings tend to encompass a greater volume of active tissue and a higher number of nodal sites, characteristics traditionally associated with greater reserve availability and the maintenance of physiological conditions required for the induction of adventitious roots [14].

From a physiological perspective, the success of adventitious rooting is associated with the efficiency of mobilization and utilization of non-structural carbohydrates and nitrogenous compounds, as well as with the adequate activity of endogenous hormonal systems, particularly those related to auxin signaling, which sustain the metabolic processes required for root induction and early development [14]. Taken together, these factors promote the differentiation of root primordia and meet the high metabolic demands characteristic of the critical rooting phase, as widely reported for vegetatively propagated species [15].

The significant effect of cutting length on adventitious rooting indicates that this parameter alone explains a portion of the observed variation in rooting performance, and is therefore a relevant morphological determinant of propagation efficiency. Such evidence reinforces that cutting length is not a marginal or secondary factor, but rather a key determinant of propagation efficiency, which can be directly manipulated in nursery management without the need for additional technological inputs.

From an applied perspective, the superior performance of 20 cm cuttings has direct implications for propagation protocols adopted by Marijuana producers and associations. Standardizing the use of longer cuttings may increase rooting uniformity, reduce propagation losses, and shorten the time required to obtain transplants ready for field establishment—since seedlings originating from cuttings carry the physiological age of the mother plant, they are therefore ready for the floral induction [16]. Although longer cuttings may reduce the total number of propagules obtained per mother plant, this disadvantage can be offset by greater rooting success and ultimately increases input efficiency and production predictability.

In this sense, the results contribute not only to the biological understanding of vegetative propagation, but also to the development of technically simple, reproducible and scalable propagation strategies, particularly relevant for small-scale growers and community-based initiatives in medicinal Marijuana.

These findings highlight cutting length as a key morphometric trait linking plant physiology, statistical evidence, and practical crop management, providing quantitative and applied support for optimizing vegetative propagation protocols in this species through the selection of cutting length.

#### 4.1.2. Cutting Fresh Mass and Rhizogenesis

The cutting fresh mass was positively associated with rhizogenic performance, as propagules with higher fresh mass exhibited superior rooting percentages compared to lighter cuttings. These results indicate that fresh mass is a biologically and operationally relevant indicator of propagation success.

Higher fresh mass reflects increased reserves of carbohydrates, amino acids, and other metabolic substrates that sustain intense cell division and differentiation during adventitious root primordia formation. These reserves act not only as energy sources but also as structural and signaling substrates [17,18].

In addition, more massive cuttings generally maintain superior water status, greater tissue hydration, and enhanced buffering capacity against dehydration stress, preserving turgor pressure and cellular integrity throughout the rooting phase [15,17]. This physiological stability favors efficient polar auxin transport and accumulation at the basal region, a central mechanism regulating adventitious root induction [19,20]. Conversely, cuttings with low fresh mass are more susceptible to rapid water loss and metabolic exhaustion, which may impair hormonal gradients and limit rhizogenic competence.

From an applied perspective, selecting cuttings with higher fresh mass can significantly improve establishment rates and produce more vigorous and homogeneous transplants, as previously reported for vegetative propagation in herbaceous and woody species [14,16].

#### 4.1.3. Basal Stem Diameter of the Cutting and Rhizogenesis

Basal stem diameter was not significantly associated with rhizogenic performance within the evaluated range, indicating limited predictive value for rooting potential in cuttings under the tested conditions.

Although thicker cuttings are often assumed to contain greater reserve pools and more developed conductive tissues [6,15], this relationship is not universal and depends on interactions among physiological status, environmental conditions, and genetic background [19,20].

The lack of association observed here suggests that variations in stem thickness do not necessarily translate into proportional gains in metabolic reserves, hormonal balance, or auxin sensitivity at the cutting base, all of which are critical for adventitious root induction [14,19].

Although basal stem diameter has traditionally been used as a proxy for cutting vigor [21], our results demonstrate that diameter alone is insufficient to discriminate cuttings with superior rhizogenic competence in this species. This finding highlights the context-dependent role of morphometric traits and underscores the need for species-specific, data-driven selection criteria rather than generalized assumptions.

Overall, these results support a definition of propagation protocols for the species, prioritizing integrated traits related to reserve status and water balance over isolated structural metrics such as stem diameter.

#### 4.1.4. Apical Stem Meristem of the Cutting and Rhizogenesis

Selecting propagules with an intact apical meristem can be considered an effective crop management strategy to enhance the success of vegetative propagation by cuttings, as consistently reported in the applied propagation literature [14,15,17,19,20]. In the present study, the maintenance of the apical stem meristem significantly improved rhizogenic performance, with cuttings retaining the apex exhibiting higher rooting rates compared to those subjected to apical removal. This result underscores the functional relevance of meristematic integrity during the early phases of adventitious root formation, rather than treating the apex as a merely morphological trait.

Physiologically, the apical meristem represents the main endogenous source of auxin biosynthesis, particularly indole-3-acetic acid (IAA), which is subsequently transported basipetally toward the cutting, where it acts as a primary signal for root primordia induction and differentiation [13,19,20]. Preserving the apex contributes to the maintenance of a stable polar auxin transport stream, ensuring continuous hormonal signaling throughout the rooting period and promoting coordinated cell division, vascular reconnection and differentiation reprogramming at the basal region [19,20].

Beyond hormonal regulation, apical integrity is also associated with higher metabolic activity and more efficient source–sink relationships, as the apex functions as an active sink that sustains carbohydrate mobilization and nitrogen allocation within the cutting [7,9]. This integrated physiological condition indirectly supports rhizogenesis by supplying energy and structural substrates required for root initiation, particularly under low-input or hormone-free propagation systems [14,20].

Although apical removal is commonly employed in mother plants to stimulate lateral branching or take several propagules per stem segment, the present results indicate that this practice may be counterproductive during the propagation phase, when rapid and reliable root induction is the primary objective.

Importantly, maintaining the apical meristem constitutes a simple, low-cost, and easily standardized management practice, applicable across cultivation scales and compatible with both conventional and agroecological production systems. This strategy is particularly relevant in production contexts where maximizing biological efficiency and minimizing reliance on external inputs are central objectives, especially in systems where the use of exogenous rooting hormones is restricted or undesirable, such as organic and or medicinal.

The observed associations reflect an integrated causal biological mechanism, in which hormonal gradients, metabolic status, and meristematic activity act synergistically to regulate adventitious rooting [20]. Thus, apical integrity is a functional and predictive determinant of propagation success, linking physiological regulation to measurable rhizogenic outcomes.

The present study provides statistically supported and physiologically coherent evidence that the presence of the apical stem meristem enhances rhizogenesis, reinforcing the inclusion of apical integrity as a key criterion in cutting-based propagation protocols, particularly in low-input or hormone-free propagation systems.

### 4.2. Colorimetry

#### 4.2.1. Variation in Cutting Color and Rhizogenesis

Variation in cutting color during the rooting phase is closely associated with physiological processes underlying rhizogenesis [22]. Changes detected through CIELAB colorimetric parameters reflect coordinated metabolic adjustments that occur during adventitious root induction, rather than random or purely senescence-related discoloration [23].

Along the A axis (green–red), the observed shift toward positive values suggests a reduction in chlorophyll content and a relative increase in secondary pigments, particularly carotenoids [24]. This pattern is consistent with the physiological response to oxidative stress commonly associated with root induction, in which carotenoids play a protective role by scavenging reactive oxygen species and stabilizing cellular membranes [20]. The statistical association between A-axis variation and rooting-related variables supports the interpretation that pigment dynamics are functionally linked to rhizogenic performance.

Changes along the B axis (blue–yellow), characterized by increased yellow reflectance, are indicative of leaf blade chlorosis, chlorophyll degradation and the mobilization of nitrogen and magnesium—key structural components of chlorophyll molecules [25]. The redistribution of these nutrients toward the basal region of the cutting is a well-documented mechanism supporting cell division and differentiation during root primordia formation [17,20,26]. In this context, the intensification of yellow coloration also reflects the accumulation of carotenoids and flavonoids, compounds associated with antioxidant defense and stress modulation during rhizogenesis [26].

Increases in luminosity (L) observed in leaves and stems at the end of the rooting period further reinforce this interpretation [24]. Tissue lightening is consistent with a decline in photosynthetic pigment concentration and the onset of controlled, partial epidermal senescence. Rather than representing tissue degradation, this process appears to facilitate the reallocation of essential nutrients—particularly nitrogen and magnesium—in favor of root development, as supported by the observed rooting outcomes [19,24].

The statistical significance of colorimetric variables highlights that CIELAB parameters function as indirect but sensitive indicators of the physiological state of propagules. These metrics integrate information on pigment composition, nutrient redistribution, and oxidative stress responses, offering a non-destructive and quantitative approach to assess rhizogenic potential.

The use of colorimetry provides growers with an innovative diagnostic tool to monitor cutting quality and rooting progression. Detecting predictable color shifts associated with successful rhizogenesis may assist in early decision-making regarding propagation timing, cutting selection, and nursery management, particularly in systems aiming to minimize chemical input.

The present results demonstrate that colorimetric variation is not merely a visual symptom, but a physiologically meaningful marker of rhizogenesis. By linking pigment dynamics, nutrient mobilization, and rooting performance, this study supports the incorporation of colorimetric analyses as a complementary approach to morphometric criteria in the optimization of vegetative propagation protocols.

#### 4.2.2. Cutting Color and Rooting Success

The foliar color is significantly associated with rooting success, supporting the interpretation of leaf chromatic attributes as indirect physiological indicators of rhizogenic competence. Logistic regression analyses consistently showed that color parameters related to leaf hue—particularly the A (green–red) and B (blue–yellow) axes—were significantly associated with rooting probability, whereas luminosity (L) and all stem colorimetric parameters exhibited no predictive relevance.

The negative association between the A axis values and rooting probability indicates that greener leaves are more likely to root successfully. This pattern suggests higher chlorophyll integrity and photosynthetic functionality at the time of cutting excision, which are essential for sustaining metabolic activity and supplying during the early stages of adventitious root formation [17,20]. Leaves with higher chlorophyll content likely maintain greater photosynthate production, supporting both energy demand and source–sink fluxes toward the basal region of the cutting [17,20].

Similarly, the observed effect of the B axis indicates that lower yellow intensity favors rooting, reinforcing the interpretation that foliar yellowing may reflect advanced nutrient remobilization or incipient senescence, conditions that can compromise rooting capacity [20,27]. While controlled nutrient export from leaves is necessary for rhizogenesis, excessive degradation of photosynthetic pigments may reduce the physiological resilience of propagules during the critical rooting phase [27].

The superior performance of multiple logistic models for leaf color, as indicated by lower AIC values, further supports the integrative nature of colorimetric traits as predictors of rooting success. The combined assessment of LAB components captures coordinated physiological processes—pigment composition, nutrient status, and metabolic activity—that are not fully explained by individual parameters alone [28]. In contrast, the absence of significant effects for stem colorimetric parameters suggests that chromatic changes associated with rhizogenesis are predominantly expressed in leaf tissues, reinforcing their role as primary source organs during vegetative propagation [17,20]

These findings highlight foliar colorimetry as a practical, non-destructive, low-cost indicator as a tool for selecting propagules with higher rooting potential that warrants further testing. The ability to discriminate rooting competence based on leaf color parameters offers clear advantages for nursery management and standardized propagation protocols, particularly in systems where minimizing chemical inputs.

This study provides statistically robust evidence that leaf colorimetric parameters—particularly those related to hue—are reliable predictors of rooting success. By linking pigment dynamics, physiological status, and rhizogenic outcomes, foliar colorimetry emerges as a complementary tool to traditional morphometric selection, reinforcing data-driven optimization of vegetative propagation protocols.

### 4.3. Morphocolorimetric Correlations of the Propagule

The Spearman correlation analysis confirmed a strong association among the propagule’s morphometric variables, indicating that such attributes represent the structural size of the propagule, a determining factor for supplying reserves for the rhizogenic process [17,20,29,30]. These results align with studies describing collinearity among morphometric parameters as an indicator of general tissue development during root induction [17,31,32].

Understanding these relationships enables the selection of cuttings with higher rooting probability, reducing losses and contributing to greater physiological and phytochemical uniformity in marijuana crops.

The integration observed between morphometric and colorimetric variables reinforces the existence of a functional relationship between structural vigor and the metabolic state of the propagule, as more robust propagules show greater pigment degradation, possibly in response to resource redistribution for rhizogenesis [15,18,20].

Thus, morphocolorimetric correlation demonstrates that rooting efficiency depends on the balance between structural reserves and metabolic homeostasis, showing that the success of vegetative propagation results from integrated processes involving morphology, physiology, and biochemistry of plant tissues [13,18,20].

The Spearman correlation analysis revealed strong and consistent associations among the propagule’s morphometric variables, indicating that cutting length, fresh mass, and related traits collectively represent overall propagule size and structural vigor. This structural dimension is a key determinant of reserve availability, directly supporting the energetic and metabolic demands of the rhizogenic.

The observed collinearity among morphometric parameters is consistent with previous reports describing coordinated tissue development during adventitious root induction, in which multiple size-related traits respond simultaneously to developmental and physiological status rather than acting independently [17,20]. Importantly, in the present study, traits that exhibited strong intercorrelations—such as cutting length and fresh mass—were also those that showed the highest predictive power for rooting success, reinforcing their functional relevance.

Beyond morphology, the integration between morphometric and colorimetric variables provides a deeper physiological interpretation of the propagation process. The correlations observed between propagule size and colorimetric shifts—particularly those related to chlorophyll degradation and increased expression of secondary pigments—indicate that more structurally robust propagules undergo more intense metabolic reprogramming during rhizogenesis. This pattern supports the interpretation that pigment degradation and chromatic variation are not just symptoms of senescence, but reflect active resource redistribution toward root formation [18,20,22,23,24].

From a statistical and applied perspective, these morphocolorimetric correlations demonstrate that rooting efficiency depends on the balance between structural reserves and metabolic homeostasis, rather than on isolated traits. The combined interpretation of morphometric and colorimetric data allows for a more accurate assessment of propagule quality and rooting potential, reducing propagation losses and contributing to greater uniformity in the crops.

The present results provide evidence that vegetative propagation success in Marijuana crops is an integrated process involving morphology, physiology and biochemistry, reinforcing the value of multivariate and correlation-based approaches for the development of species-specific, efficient, and reproducible propagation protocols.

## 5. Conclusions

**Figure 8:**
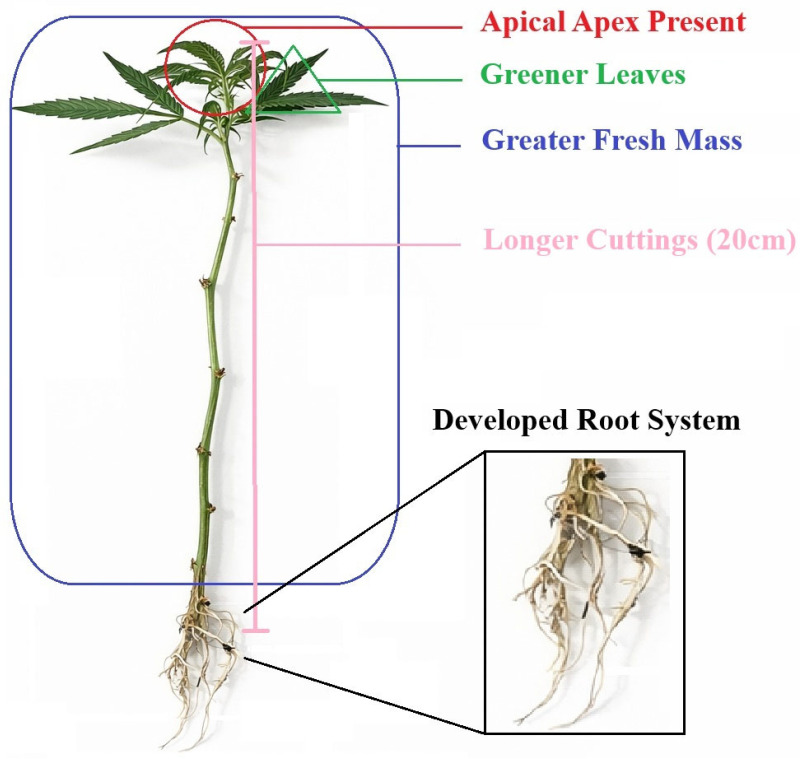
Schematic representation of the morphological and colorimetric traits associated with successful vegetative propagation of Marijuana (*Cannabis sativa* L.) Cuttings exhibiting the presence of the apical apex, greener leaves, greater fresh mass, and increased cutting length (20 cm) showed higher rooting probability, resulting in a developed root system after 21 days of propagation.

Vegetative propagation of marijuana (*Cannabis sativa* L.), evaluated under semi-controlled conditions using the cultivar ‘Doctor’, was significantly influenced by set of morphometric and foliar colorimetric attributes of the propagule. Cuttings with greater length (20 cm), higher fresh mass, and the presence of an apical meristem exhibited a higher probability of successful rhizogenesis, while leaf colorimetric parameters indicated that greener leaves and reduced yellowing were associated with improved outcomes. In contrast, basal stem diameter and stem color were not predictive of rooting success (Figure 8).

Importantly, the measured traits explained only part of the variability in rooting performance, indicating that rhizogenic success is influenced by additional factors, including unmeasured environmental variation. Therefore, while these characteristics can serve as useful selection criteria, standardization of mother plant management and propagation chamber conditions (e.g., temperature, humidity, and light) may be relevant for achieving optimized rooting rates.

In this context, foliar colorimetry emerges as a non-destructive, low-cost, quantitative indicator of physiological status, particularly useful for monitoring rooting dynamics and supporting decision-making in low-input propagation systems. The absence of predictive value for stem color and basal stem diameter further refines phytotechnical criteria by empirically excluding non-informative parameters.

From an applied perspective, adopting these morphometric and colorimetric attributes as selection criteria may reduce propagation losses, improving uniformity and rationalizing input use, especially in community-based production systems or those operating under limited environmental control. Overall, this study contributes to the technical basis for sustainable, evidence-based vegetative propagation of marijuana by cuttings, while future research should validate these associations across multiple genotypes and under controlled conditions to assess their broader applicability.

## Figures and Tables

**Table 1:** The analysed variables of the propagules, equipment used, and respective collection moments during the vegetative propagation experiment of marijuana (*Cannabis sativa* L.) cutting.

Variable	Equipment	Moment ^1^
Length	Ruler	Initial
2. Fresh mass	Precision scale	Initial
3. Diameter of the stem base	Digital caliper	Initial
4. Apical shoot meristem	Cutting blade	Initial
5. Stem color	Colorimeter	Initial and Final
6. Leaf color	Colorimeter	Initial and Final
7. Number of root primordia	Magnifying glass	Final
8. Size of adventitious roots	Magnifying glass and digital caliper	Final

^1^ Initial: Cutting from the mother plant; Final: The 21st day after cutting.

**Table 2:** Statistical strategy and the objectives, variables, and statistical methods of marijuana (*Cannabis sativa* L.) cuttings.

Objective	Variables	Response	Statistical Method
Morphometry	Length	Rhizogenesis	Simple and contrast logistic regression
	Fresh mass	Rhizogenesis	Simple logistic regression
	Diameter of the stem base	Rhizogenesis	Simple logistic regression
	Stem apical meristem	Rhizogenesis	Binary logistic regression; Fisher; χ^2^
Colorimetry	Initial colors of stems and leaves	Final colors of stems and leaves	Mean *t*-test
	Initial colors of stems and leaves	Rhizogenesis	Simple and multiple logistic regression
Correlations	Length Fresh mass Diameter of the stem base Stem apical meristem Initial colors of stems and leaves Final colors of stems and leaves Number of root primordia Size of adventitious roots		Spearman Correlation Matrix; Principal Component Analysis (PCA).

**Table 3:** Means and standard deviations (±SD) of the morphological variables of propagules at different lengths (5, 10, 15, and 20 cm) of marijuana (*Cannabis sativa* L.), including survival and rooting rates (%), fresh mass (g), basal stem diameter (mm), number of root primordia, and length of adventitious roots (mm).

Variable	5 cm	10 cm	15 cm	20 cm	Total
Survival (%)	67	77	67	87	74
Rooting (%)	38	33	32	53	39
Mass (g)	0.60 ± 0.31	0.81 ± 0.31	1.35 ± 0.35	2.40 ± 0.43	1.30 ± 0.78
Diameter (mm)	2.39 ± 0.34	2.68 ± 0.42	3.69 ± 0.92	4.23 ± 0.79	3.25 ± 1.00
Primordia (#)	6.24 ± 9.87	2.09 ± 5.55	6.12 ± 10.42	7.75 ± 10.40	5.58 ± 9.45
Roots (mm)	13.17 ± 23.10	4.32 ± 13.82	16.58 ± 33.04	23.48 ± 30.92	14.62 ± 27.12

# indicates the number of root primordia.

**Table 4:** Simple and multiple logistic regression models adjusted for the color components of leaves and stems, as predictors of rooting in marijuana (*Cannabis sativa* L.) propagules, with 90% confidence intervals (CI_90_).

Model	Plant Part	CIE-LAB	Estimative	IC_90_	*p*-Value	AIC
Simple	Leaf	L	1.000	0.942–1.070	0.913	325
		A	0.870	0.752–0.985	0.089 *	322
		B	0.988	0.922–1.060	0.767	325
	Stem	L	0.988	0.961–1.020	0.489	323
		A	0.966	0.923–1.010	0.215	322
		B	1.010	0.978–1.040	0.607	323
Multiple	Leaf	LAB	1.080	0.996–1.170	0.117	320
		LAB	0.727	0.590–0.874	0.008 *	320
		LAB	0.894	0.800–0.985	0.077 *	320
	Stem	LAB	1.000	0.964–1.040	0.907	326
		LAB	0.966	0.913–1.020	0.298	326
		LAB	1.000	0.965–1.050	0.845	326

* indicates statistically significant at *p* < 0.10.

**Figure A1:**
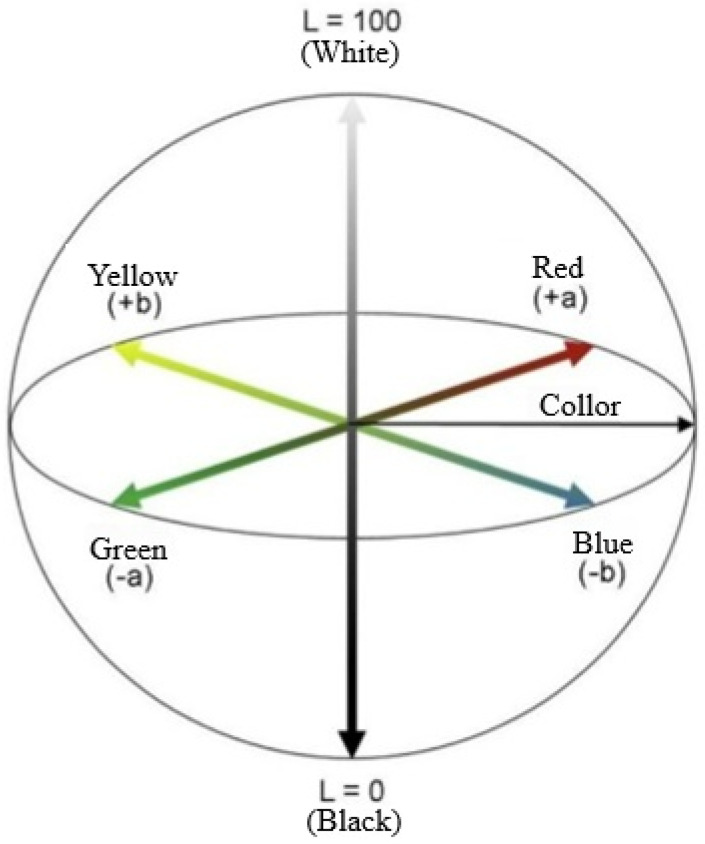
Schematic representation of the CIELAB color space, illustrating the three-dimensional axes used for color characterization. The L axis represents lightness, ranging from black (L = 0) to white (L = 100), while the A axis spans from green (−A) to red (+A), and the B axis from blue (−B) to yellow (+B). This color model was used to quantify color attributes in the study.

**Figure A2:**
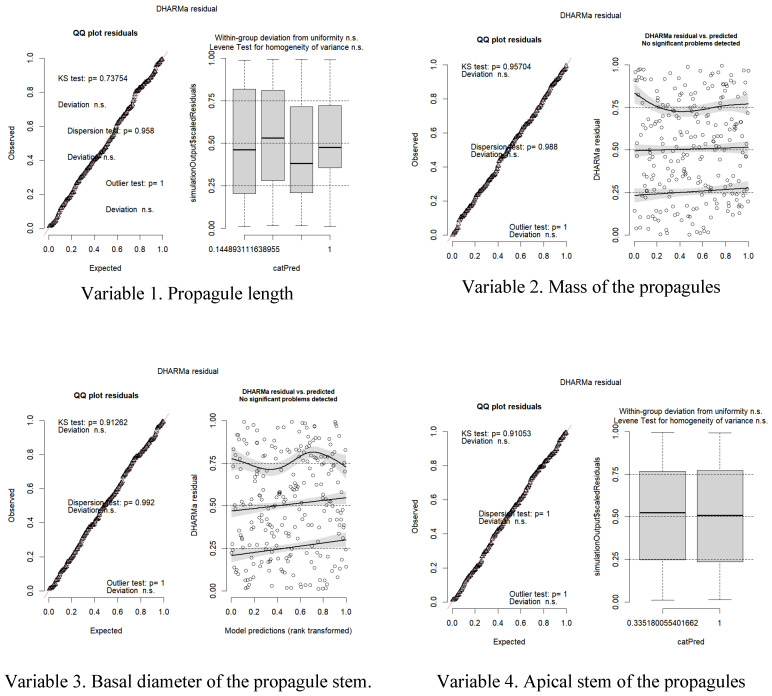
Diagnostic plots from DHARMa residual analyses for generalized linear models evaluating the effects of propagule traits on rooting responses. Panels correspond to (Variable 1) propagule length, (Variable 2) propagule mass, (Variable 3) basal diameter of the propagule stem, and (Variable 4) apical stem diameter. For each variable, quantile–quantile (QQ) plots of simulated residuals, residuals versus predicted values, and dispersion and homogeneity tests are presented, indicating overall model adequacy and absence of major deviations from distributional assumptions.

## Data Availability

The original contributions presented in this study are included in the article. Further inquiries can be directed to the corresponding author(s).

## Appendix A

The Representation of colorimetry in the CIELAB system.

## Appendix B

Evaluation of model assumptions through the analysis of standardized simulated residuals using the DHARMa package. The plots show, from left to right: (i) QQ plot of the simulated residuals compared to the expected theoretical distribution, with non-significant Kolmogorov–Smirnov (KS), dispersion, and outlier tests, indicating no relevant deviations in the residual distribution; (ii) boxplots of residuals by categories of the predictor, with non-significant results in the tests for homogeneity of variance (Levene) and deviation from uniformity within groups. The results indicate that the evaluated models adequately meet the assumptions of normality, homoscedasticity, and independence of residuals in the data from the marijuana (*Cannabis sativa* L.) cutting experiment.
